# Engineered extracellular matrices with controlled mechanics modulate renal proximal tubular cell epithelialization

**DOI:** 10.1371/journal.pone.0181085

**Published:** 2017-07-17

**Authors:** Jeffrey A. Beamish, Evan Chen, Andrew J. Putnam

**Affiliations:** 1 Division of Nephrology, Department of Internal Medicine, University of Michigan, Ann Arbor, Michigan, United States of America; 2 Department of Biomedical Engineering, University of Michigan, Ann Arbor, Michigan, United States of America; National Cancer Institute, UNITED STATES

## Abstract

Acute kidney injury (AKI) is common and associated with significant morbidity and mortality. Recovery from many forms of AKI involves the proliferation of renal proximal tubular epithelial cells (RPTECs), but the influence of the microenvironment in which this recovery occurs remains poorly understood. Here we report the development of a poly(ethylene glycol) (PEG) hydrogel platform to study the influence of substrate mechanical properties on the proliferation of human RPTECs as a model for recovery from AKI. PEG diacrylate based hydrogels were generated with orthogonal control of mechanics and cell-substrate interactions. Using this platform, we found that increased substrate stiffness promotes RPTEC spreading and proliferation. RPTECs showed similar degrees of apoptosis and Yes-associated protein (YAP) nuclear localization regardless of stiffness, suggesting these were not key mediators of the effect. However, focal adhesion formation, cytoskeletal organization, focal adhesion kinase (FAK) activation, and extracellular signal-regulated kinase (ERK) activation were all enhanced with increasing substrate stiffness. Inhibition of ERK activation substantially attenuated the effect of stiffness on proliferation. In long-term culture, hydrogel stiffness promoted the formation of more complete epithelial monolayers with tight junctions, cell polarity, and an organized basement membrane. These data suggest that increased stiffness potentially may have beneficial consequences for the renal tubular epithelium during recovery from AKI.

## Introduction

Acute kidney injury (AKI) is common, costly, and associated with increased mortality [1–3]. Many forms of AKI are reversible and often involve the regeneration of damaged renal tubular epithelium. There is great interest in better understanding factors that influence renal tubular epithelial regeneration to mitigate the short-term and long-term consequences of AKI in terms of patient health and societal costs.

Patients with pre-existing chronic kidney disease (CKD) who develop AKI are more likely to progress to end stage renal disease (ESRD) [4]. Interstitial fibrosis is the final common pathway in most forms of CKD and is associated with a worse prognosis [5]. Such fibrosis is accompanied by tissue stiffening. Use of magnetic resonance and sonographic techniques to measure tissue mechanical properties as a surrogate for fibrosis is attracting interest as a non-invasive diagnostic approach in renal transplants [6] and other organs [7]. Using these types of imaging techniques, it has been observed that even patients with relatively mild CKD have increased stiffness of the renal parenchyma [8,9]. How this increased parenchymal stiffness might impact recovery from AKI remains poorly understood.

Extracellular matrix (ECM) mechanics influence the behavior and differentiation of a wide range of cell types in physiologic and pathologic circumstances [10,11], and specifically, matrix mechanics have been implicated as a key modulator of cell proliferation [12]. Renal proximal tubular epithelial cell (RPTEC) proliferation is central to recovery from many forms of AKI. After injury, the renal epithelium undergoes apoptotic and necrotic death followed by proliferation of surviving RPTECs at sites of injury and culminates with regeneration of a functional epithelium [13–15]. The nature of this repopulation suggests that the denuded basement membrane left behind by injured epithelial cells is instructive in the regeneration process.

While much is known about the multitude of biochemical and cellular modulators of AKI recovery (reviewed [16]), relatively little is known about the role of basement membrane mechanics on this process. In an *in vitro* model using primary mouse RPTECs, cells cultured on soft substrates have delayed upregulation of cyclin D1 early after plating and resist transforming growth factor β1 induced epithelial-to-mesenchymal transition [17]. Factors implicated as key mediators of mechanical signal transduction *in vitro*, such as focal adhesion kinase (FAK), extracellular signal-regulated kinase (ERK), and Yes-associated protein-1 (YAP) [11,18], are upregulated or activated after AKI *in vivo* [19,20].

Recently, there has been revived interest in generalized regenerative strategies for the kidney [21], which could benefit both patients with AKI and ESRD. Developing scaffolding systems that guide regeneration remains a critical barrier to progress. Control of scaffold mechanics is widely recognized as a key parameter in scaffold design [22]. To date, systems used to study the effects of mechanics on RPTECs are either toxic (such as polyacrylamide) or have inherently entangled biochemical and mechanical properties (such as collagen or Matrigel). Poly(ethylene glycol) (PEG) based hydrogels can be easily tailored to generate a wide range of mechanical moduli, can more effectively disentangle the effects of ECM biochemistry from mechanics [11,23,24], and have been used widely as a platform for a variety of regenerative strategies [25].

Here we report the development of a synthetic basement membrane with isolated mechanical properties using a PEG-based hydrogel platform. Using this system as an *in vitro* model, we explore the mechanisms by which substrate mechanics influence the regenerative potential of renal tubular epithelium of human origin.

## Materials and methods

### Reagents

Unless noted, all chemical reagents were obtained from Sigma (St. Louis, MO) and all cell culture reagents were obtained from Thermo Fisher Scientific (Waltham, MA).

### Poly(ethylene glycol) diacrylate synthesis

PEG (MW 3400) was dried by azeotropic distillation with toluene and cooled to room temperature. Under argon, acryloyl chloride (2 molar excess per OH), then dry triethylamine (4.4 molar excess per OH), then additional acryloyl chloride (2 molar excess per OH, 4-fold molar excess total) were added dropwise and the reaction was stirred overnight at room temperature. The resulting suspension was filtered, precipitated in ice-cold diethyl ether, and dried *in vacuo*. This crude product was dissolved in a minimal volume of dichloromethane, filtered, precipitated in ice-cold diethyl ether, and dried *in vacuo*. The resulting PEG diacrylate (PEGDA) product was stored under argon at -20°C until use. Substitution was approximately 86%, as determined by ^1^H NMR.

### Fabrication and characterization of synthetic ECMs

Hydrogel precursor solutions were prepared with various weight compositions of PEGDA in phosphate buffered saline (PBS) and 1 mg/ml Irgacure 2959 (Ciba, Basel, Switzerland) as previously described [23]. Drops of precursor solution were placed on a fluorosilanized glass plate, covered with 22 mm glass coverslips functionalized with 3-(Trimethoxysilyl)propyl methacrylate, and polymerized with UV light (10 min, 365 nm, 6 mW/cm^2^) to form hydrogel films approximately 150 μm thick. The resulting gel-glass composite substrates were covered with ethanol, gently pried from the larger glass plate with a razor (still attached to the coverslip on the other side), inverted, and transferred to PBS overnight.

To provide cell-gel interactions, collagen IV was conjugated to the hydrogel surface. To do this, substrates were first incubated in MES buffer (0.1 M 2-[N-morpholino]ethanesulfonic acid, 0.5 M sodium chloride, pH 6) for 30 min, the buffer was exchanged for 0.25 mg/ml sulfoSANPAH (Proteochem, Hurricane, UT) in MES buffer, and substrates were exposed to UV light (16 min, 365 nm, 3 mW/cm^2^). Substrates were rinsed briefly with MES and the sulfoSANPAH treatment was repeated. The substrates were rinsed briefly with MES for 5 min then briefly with HEPES buffer (0.5 M 4-[2-hydroxyethyl]-1-piperazineethanesulfonic acid, pH 9.0), incubated with 25 μg/ml collagen type IV from human placenta (Collagen IV, Sigma) in HEPES buffer on a rocker for 2.5 h, and then with 0.5 M ethanolamine in HEPES buffer for 60 min to quench unreacted sulfoSANPAH functionality. Substrates were rinsed briefly with PBS followed by 4 additional rinses of at least 30 min in duration. Collagen IV also was adsorbed on ethanol-disinfected bare glass coverslips overnight at 4°C and rinsed with PBS twice prior to use.

Conjugation of collagen IV onto the gel surface was quantified using immunofluorescence staining for collagen IV (S1 Table) using an Alexa Fluor 568 goat-anti-mouse secondary antibody (1:400, Thermo Fisher) for detection and compared with background staining on samples not exposed to collagen IV during conjugation but otherwise processed identically. At each location in a predefined 3 x 3 grid, the focal plane of the gel surface was located by phase contrast microscopy, then epifluorescence images were acquired with fixed exposure and acquisition settings. This process was repeated for all samples in each experiment. Average pixel intensity and distribution were quantified using ImageJ [26].

To measure hydrogel moduli, 0.8 mm thick hydrogel slabs were formed between glass plates, incubated with PBS overnight, MES buffer for 2h, HEPES buffer for 2.5h, HEPES buffer with 0.5 M ethanolamine for 60 min, and then several PBS rinses (to simulate Collagen IV conjugation to the hydrogel films). Cylinders (8 mm diameter) were punched from these slabs and the shear storage modulus was measured over a 3 minute time sweep at 0.5 Hz, 0.05% strain, and 0.05 N normal force for 3 samples for each of 3 independent trials using an AR G2 rheometer (TA Instruments, New Castle, DE). 3.50% PEGDA gels were too weak to survive additional PBS rinses, so rheology was performed immediately after incubation with ethanolamine and using 5% strain and a fixed plate separation of 0.5 mm. Given the low moduli of the 3.50% samples, substantially increased strain was required to achieve an adequate signal-to-noise ratio during the measurement.

### Cell culture

Human RPTECs were obtained from Lonza and cultured in renal epithelial growth media (REGM, Lonza, Walkersville, MD) that includes 0.5% fetal bovine serum (FBS) and human epidermal growth factor among other supplements. The manufacturer confirms positive staining for gamma-glutamyltransferase, which is normally expressed in mature proximal tubular epithelium, prior to shipment. We further demonstrated epithelial phenotype by confirming cell-cell junction expression of Zo-1 and a single central cilium (detected as a punctate focus of acetylated tubulin staining) at confluence. RPTECs were expanded, cryopreserved, and then used for experiments between passage 4–6 except for cell attachment studies that included cells up to passage 8. RPTECs were detached from routine culture flasks with 0.025% trypsin (Lonza), then mixed with trypsin neutralizing solution (Lonza), pelleted, and resuspended in REGM to plate on substrates at 10,000 cells/cm^2^ for growth, attachment, and immunofluorescence studies and 30,000 cells/cm^2^ for protein isolations done at early time points. In some experiments, RPTECs were seeded on tissue culture plastic and treated with staurosporine (Sigma) to induce apoptosis as a positive control for caspase 3 and DAPI apoptosis assays. In other experiments, RPTECs were cultured in medium containing PD98059 (range: 1–100 μM, 50 μM for experiments on hydrogel substrates, Thermo Fisher) or dimethyl sulfoxide (DMSO) which was made fresh and exchanged daily.

Normal human lung fibroblasts (NHLFs, Lonza) were plated and cultured in M-199 medium supplemented with 10% FBS, used between passage 8–14, and plated at the same densities as RPTECs. Human mesenchymal stem cells (MSCs, RoosterBio, Frederick, MD) were cultured in high glucose Dulbecco's modified Eagle's medium (DMEM) supplemented with 10% FBS, used at passage 6, and plated on substrates at 6,000 cells/cm^2^.

### Microscopy and immunofluorescence staining

Phase contrast images were acquired just prior to fixation. Cells were rinsed with PBS, fixed with 4% paraformaldehyde in PBS for 10 min at room temperature, permeabilized with 0.1% Triton X-100 for 10 min, incubated with 1 μM DAPI for 30 min, and imaged. These images were used for cell density quantification (to prevent cell loss during subsequent processing steps). For many samples, additional staining was performed. These samples were blocked in tris-buffered saline with 0.1% Tween-20 (TBST) with 2% bovine serum albumin, incubated with primary antibody (S1 Table) for 60 min at room temperature, rinsed twice with TBST, incubated with isotype appropriate Alexa Fluor 488 or 568 goat-anti-mouse secondary antibodies (1:200–1:400, Thermo Fisher), Alexa Fluor 488 phalloidin (1:200, Thermo Fisher), and 1 μM DAPI for 45 min, and rinsed twice with TBST. Care was taken to minimize the time gel substrates were not submerged in buffer. Where high magnification imaging was needed, substrates were placed on glass slides gel side up, then coverslips (24x40 mm) were placed on the samples over anti-fade mounting medium, clamped gently down onto the gel, and sealed with epoxy prior to imaging. Epifluorescence images were acquired using an Olympus IX81 microscope (Center Valley, PA). Z-stack images were acquired on a Nikon A-1 confocal microscope (Melville, NY).

### Image analysis

Cell area was quantified using ImageJ for 45 cells at prespecified locations from 5 phase contrast images using 10x original magnification. Area coverage was determined by thresholding 5 phalloidin stained 4x images in a manner that optimally separated the background and phalloidin signals within a circular region of interest in each image. DAPI stained nuclei were quantified from 10x images using ImageJ for 9 images per condition. Nuclear distribution of YAP was determined by a blinded observer for all nuclei contained in 9 images acquired at 20x. DAPI images taken in parallel were used to define the nuclei in these experiments. Semi-quantitative analysis of stress fibers and focal adhesions was performed on 12 randomly selected cells at 60x magnification. Focal adhesions were defined as punctate areas of vinculin staining distinct from the surrounding background with a minor dimension (to allow for the inclusion of elongated focal adhesions) of 0.7 to 4.3 μm. All images were acquired at prespecified locations, except for 60x images where artifacts induced by the mounting process limited this approach. Each of the analyses described above were repeated for each condition in each of 3 independent experiments.

### Immunoblotting

Cultures were rinsed with PBS then incubated with ice-cold radioimmunoprecipitation assay buffer (Santa Cruz Biotechnology, Dallas, TX supplemented with protease and phosphatase inhibitors included in the kit as well as 5 mM sodium fluoride) for 20 min. Cells were gently scraped from the surfaces of the substrates with a rubber spatula (destroying the softer gels). Resulting mixtures were centrifuged at >15,000*g* for 5 min at 4°C, the supernatant removed attempting to avoid hydrogel particles, a portion mixed with gel loading buffer, and the samples boiled for 5 min. Total protein content was determined by bicinchoninic acid assay (Thermo Fisher). Protein (3 μg per sample) was loaded and subjected to sodium dodecyl sulfate polyacrylamide gel electrophoresis and transferred to a polyvinylidene difluoride membrane. In some experiments, 5–12.5 μg whole human kidney lysate protein (Santa Cruz Biotechnology) was loaded as a control. Blots were probed with indicated primary antibody (S1 Table) followed by species appropriate horseradish peroxidase-conjugated secondary antibody and developed using enhanced chemiluminescence. In several cases, membranes were subsequently stripped using mild stripping buffer (AbCAM, Cambridge, MA) and reprobed. Reprobed membranes were spot checked to ensure minimal residual signal observed after this protocol. Resulting films were scanned at high resolution and densitometry was performed using ImageJ.

### Statistics

Statistical analysis was performed using GraphPAD Prism. Data are represented as mean ± standard deviation of at least 3 independent experiments. Continuous (or approximated continuous) data were analyzed using ANOVA with Tukey *post-hoc* testing or, in some cases, ANOVA with *post-hoc* trend analysis which assessed whether group means increased or decreased systematically with substrate stiffness. Categorical data was analyzed by χ^2^ contingency table analysis. A value of α < 0.05 was considered significant.

## Results

### Substrate mechanics are independent of ECM conjugation

**Fig 1:**
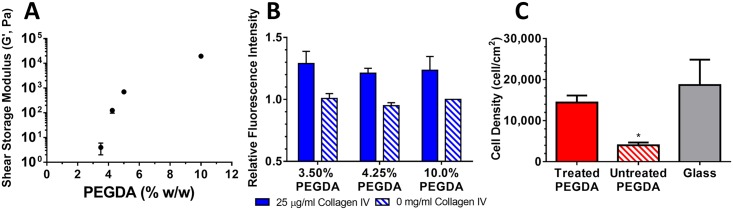
Characterization of PEGDA hydrogel based synthetic ECMs. (A) A wide range of mechanical properties was achieved by varying the mass fraction of PEGDA at polymerization from 3.50% to 10.0% (w/w). The shear storage modulus at 0.5 Hz, 0.05% strain was measured using a parallel plate rheometer. (B) Collagen IV conjugation does not depend on gel composition. Conjugation of collagen IV was quantified using the relative fluorescence signal after immunofluorescence staining (solid bars) and compared with gels processed identically but without exposure to collagen IV (hashed bars). (C) Surface functionalization is needed to support robust cell attachment. 10% PEGDA gels with and without sulfoSANPAH mediated conjugation of collagen IV were plated with RPTECs. After 4 h surfaces were rinsed, fixed, and adherent cell density quantified by counting DAPI stained nuclei. *: *p* < 0.05 relative to treated PEGDA and glass.

We systematically generated hydrogel substrates with moduli above and below the estimated shear modulus of the renal parenchyma measured sonographically, which is about 1–2 kPa in the normal kidney [9]. Because others have shown a tight correlation between bulk hydrogel properties and the mechanics of thin hydrogel films [27], we measured the rheological properties of bulk hydrogel slabs after simulated surface modification. Shear storage moduli were 4 ± 2 Pa, 0.13 ± 0.03 kPa, 0.70 ± 0.03 kPa, and 19 ± 0.6 kPa for 3.50%, 4.25%, 5.00% and 10.0% (w/w) PEGDA gels, respectively (Fig 1A). The measured shear loss moduli for the samples were 1 ± 1 Pa, 20 ± 16 Pa, 37 ± 20 Pa, and 1.4 ± 0.06 kPa for each composition respectively, indicating minimal viscous contribution to the properties at low frequencies.

To confirm that cell response to the synthetic ECMs was due to differences in mechanics and not due to differences in the conjugation of ECM proteins, we quantified collagen IV conjugation using immunofluorescence staining. The relative fluorescence intensity was similar on all collagen IV conjugated hydrogels with no statistical differences measured using this technique (Fig 1B). As has been previously observed with other ECM proteins [11], surface distribution of collagen IV was inhomogeneous (S1A Fig). However, when analyzed in a fashion similar to Wen *et al*. [28], the distribution of pixel intensities was similar between collagen IV conjugated hydrogels, again suggestive of similar surface conjugation (S1B Fig).

Surface modification was necessary to allow cell attachment to the gels. RPTECs failed to attach robustly to unmodified 10% PEGDA substrates (Fig 1C). However, RPTEC attachment to collagen IV conjugated hydrogels was similar to collagen IV coated glass. The few cells that remained on the unmodified PEGDA substrates were poorly spread and rounded in morphology, while the appearances of cells on the modified hydrogels and glass were similar.

### Stiffness facilitates RPTEC spreading and proliferation

**Fig 2:**
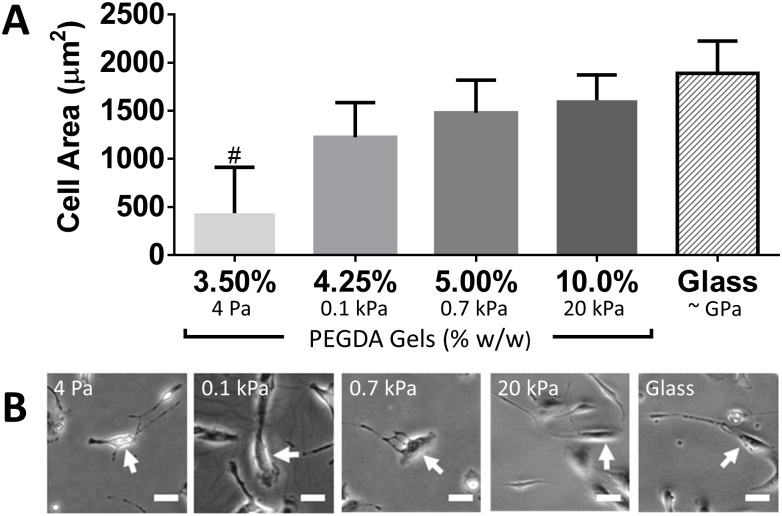
Stiff matrix promotes RPTEC cell spreading. (A) RPTECS were plated at 10,000 cells/cm^2^ and allowed to attach for 24 h. Prior to washing, cell area was manually quantified from phase contrast images for 45 cells at predetermined locations in each of 3 independent trials. (B) Phase contrast images of individual cells with measured cell area closest to the group mean. ANOVA with *post-hoc* trend analysis for cell area was significant (*p* < 0.05) for trend from 4.25% (0.1 kPa) to Glass. #: *p* < 0.05 relative to all other substrates. Percentages indicate composition of PEGDA at polymerization (% w/w) for hydrogel substrates. Scale bar: 25 μm (applies to all images).

**Fig 3:**
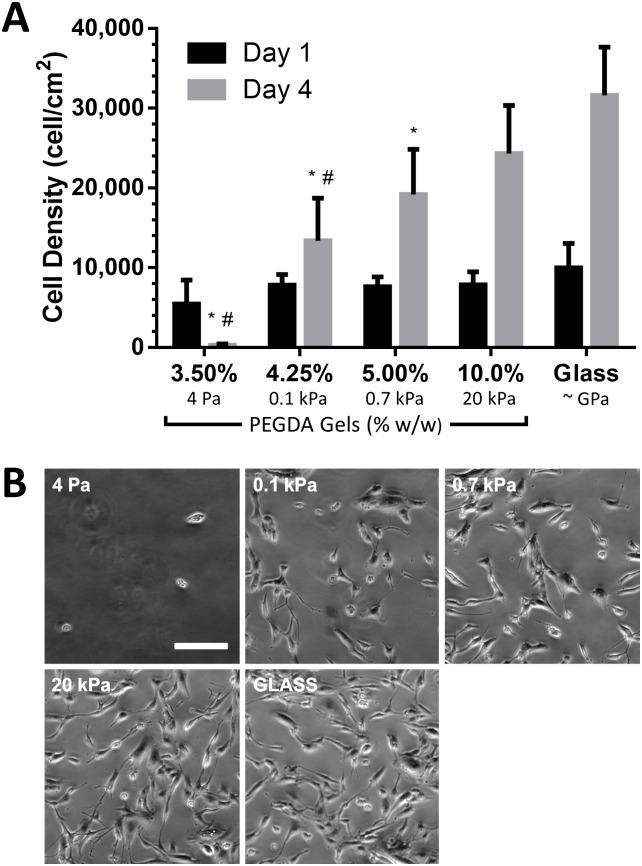
Stiff matrix promotes RPTEC proliferation. (A) RPTECS were plated at 10,000 cells/cm^2^ and cultured for 4 d. At 1 d or 4 d, cell density was quantified by counting DAPI stained nuclei. (B) Phase contrast images showing cell morphology 4 d after plating. *: *p* < 0.05 relative to glass; #: *p* < 0.05 relative to 10.0% (20 kPa). Percentages indicate composition of PEGDA at polymerization (% w/w) for hydrogel substrates. Scale bar: 200 μm (applies to all images).

As a model of recovery after AKI, RPTECs were plated on synthetic ECMs of varying stiffness and cultured in REGM to drive cell proliferation. One day after plating, we noted that cell area on the 3.50% (4 Pa) PEGDA gels was significantly smaller than the other substrates (Fig 2A). For the remaining gel compositions, we noted a significant correlation between area and substrate stiffness (*p* < 0.05) though cell morphology was similar between substrates (Fig 2B). One day after plating, cell density was 5440 ± 3020, 7820 ± 1340, 7600 ± 1240, 7840 ± 1660 on the 3.50% (4 Pa), 4.25% (0.1 kPa), 5.00% (0.7 kPa), 10.0% (20 kPa) PEGDA synthetic ECMs respectively and 9970 ± 3090 cells/cm^2^ (target plating density 10,000 cells/cm^2^) on collagen IV coated glass (Fig 3A). Over the following 3 d, cell number increased by 1.7-fold on 4.25% (0.1 kPa) PEGDA gels compared with 3.1-fold and 3.2-fold increases on 10.0% (20 kPa) PEGDA and glass respectively. By 4 d, most cells on 3.50% (4 Pa) PEGDA gels detached. Aside from the 3.50% (4 Pa) PEGDA gels, RPTEC morphology at 4 d was similar on all substrates (Fig 3B).

### Apoptosis does not explain differences in cell density

**Fig 4:**
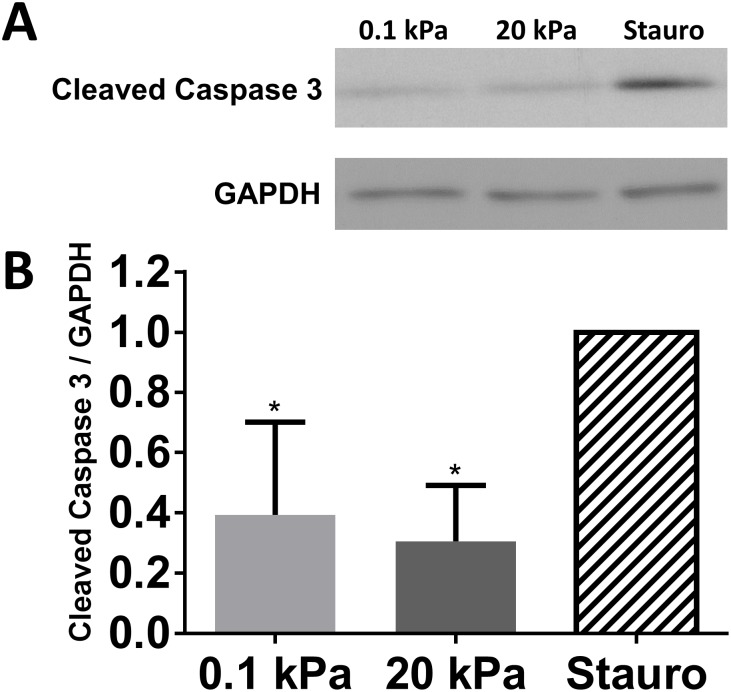
Matrix stiffness does not affect RPTEC apoptosis. (A) RPTECs were allowed to attach to substrates for 24 h, then cells were exposed to fresh medium (hydrogel substrates) or medium with 2 μM staurosporine (Stauro) for an additional 24 h. Levels of cleaved caspase 3 were determined by western blot. (B) densitometry. *: *p* < 0.05 relative to staurosporine treated controls.

As substrate mechanics have been linked to differences in apoptosis rates [29], and could contribute to differences in cell density, we evaluated whether apoptosis early in culture was contributing to the observed differences in cell number. After 2 d in culture we observed low levels of cleaved caspase 3 that were similar between 0.1 kPa and 20 kPa gels, but lower than controls treated with staurosporine, which induces apoptosis and served as a positive control (Fig 4). We also examined DAPI-stained nuclei 1–2 d after plating and noted few cells with definitive nuclear fragmentation [30] (as seen in staurosporine-treated controls) suggesting negligible apoptosis regardless of substrate (S2 Fig).

### Stiffness modestly modulates YAP nuclear localization

**Fig 5:**
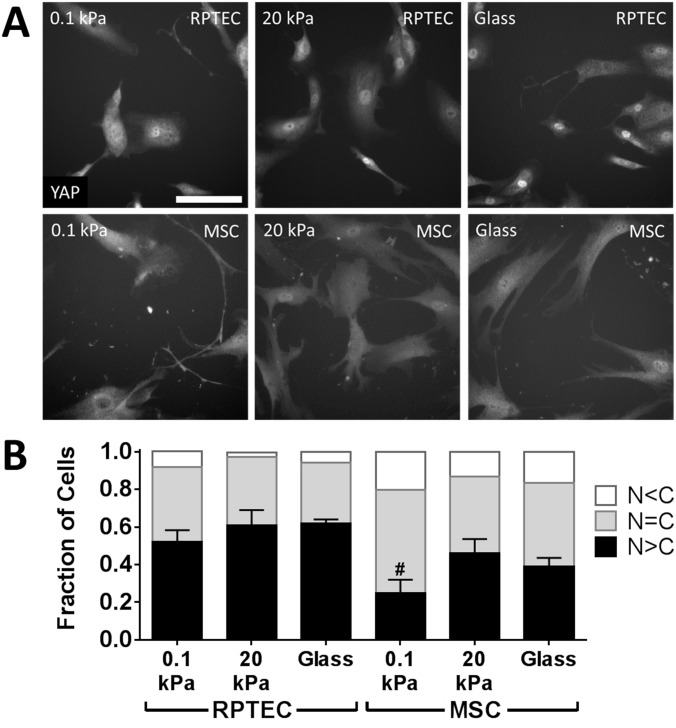
Matrix stiffness modestly affects YAP nuclear localization. (A) RPTECs were allowed to attach to substrates for 24 h, then were fixed and stained for YAP. All cells in each of 9 images per condition per experiment were categorized as having stronger (N>C), similar (N = C), or weaker (N<C) YAP staining in the nucleus than the cytoplasm. Mesenchymal stem cells (MSCs), which have a well-described alteration in YAP nuclear localization with substrate stiffness, were used as a control. Images with distributions most closely matching the overall mean are shown. (B) distributions of YAP localization for RPTECs and MSCs. χ^2^ contingency table analysis for RPTECs or MSCs (analyzed separately) yields a distribution dependence on substrate with *p* = 0.006 and *p* = 0.0005, respectively. Treating the fraction of cells with N>C as a continuous variable with ANOVA and Tukey’s *post-hoc* test shows only a difference between MSCs on 0.1 kPa vs 20 kPa gels (#, *p* < 0.05). Scale bar: 100 μm (applies to all images).

YAP nuclear localization has been proposed as a key mediator of substrate stiffness effects [31]. We observed that 24 h after plating YAP nuclear localization was high and relatively insensitive to substrate stiffness in RPTECs compared with MSCs, which showed significant differences depending on substrate stiffness and served as controls (Fig 5). After 48 h, nearly all RPTECs had predominantly nuclear localized YAP (S3 Fig).

### Stiffness enhances cytoskeletal organization

**Fig 6:**
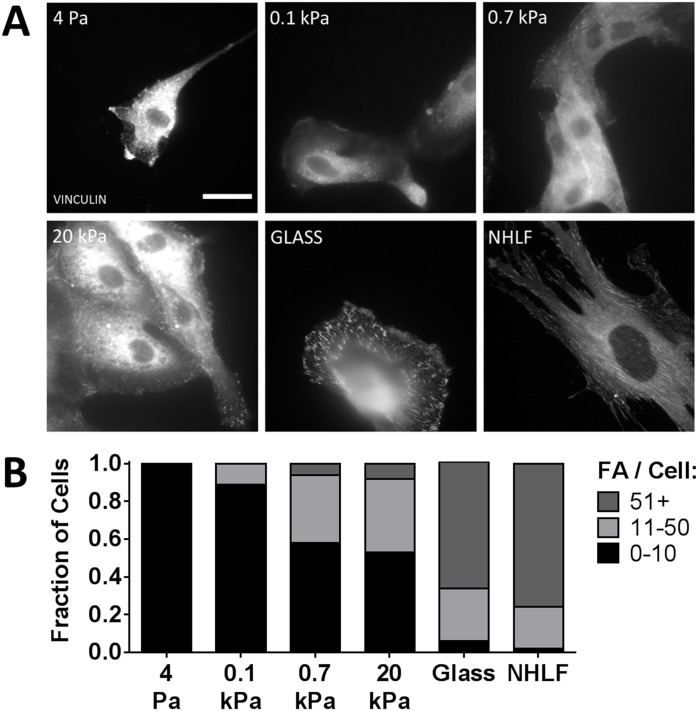
Increasing substrate stiffness promotes vinculin-containing focal adhesion formation. (A) Focal adhesions (FA) were identified using immunofluorescent staining for vinculin (grayscale) 24 h after plating. (B) Randomly selected cells were categorized by number of focal adhesions per cell. χ^2^ contingency table analysis for RPTECs (NHLF data excluded from analysis) was highly significant for substrate effect on distribution, *p* < 0.0001. Scale bar: 25 μm (applies to all images).

As cytoskeletal organization previously has been associated with stiffness-dependent modulation of cell proliferation [32], we evaluated both the development of focal adhesions and actin stress fiber formation in our model. The assembly of focal adhesions, identified by the presence of vinculin, exhibited a marked dependence on substrate stiffness. On 4 Pa and 0.1 kPa gels, very few vinculin-containing focal adhesions were clearly identified (Fig 6A). As substrate stiffness increased further, vinculin-containing adhesions became more prominent. However, even on glass substrate controls, the number of focal adhesions identified in each RPTEC varied widely, in contrast to fibroblast controls (NHLFs) in which essentially all cells expressed robust focal adhesions. To better analyze these distributions, randomly selected cells were assigned to categories of minimal (0–10), moderate (11–50), or extensive (50+) vinculin containing focal adhesions (Fig 6B). We observed a clear shift in the distribution of these focal adhesion phenotypes as substrate stiffness increased.

**Fig 7:**
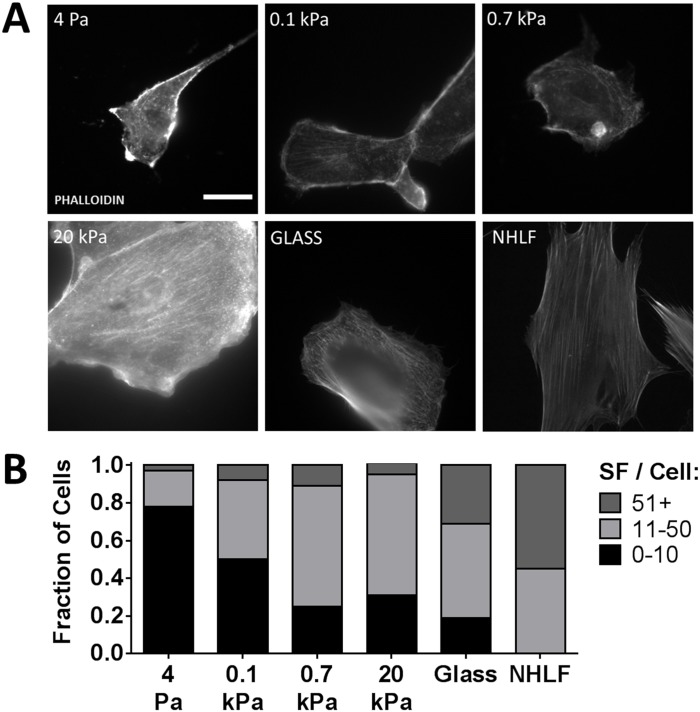
Increasing substrate stiffness promotes stress fiber formation. (A) Filamentous actin was identified with fluorescently tagged phalloidin (grayscale) 24 h after plating. (B) Randomly selected cells were categorized by number of well-developed central stress fibers (SF) per cell. χ^2^ contingency table analysis for RPTECs (NHLF data excluded from analysis) was highly significant for substrate effect on distribution, *p* < 0.0001. Scale bar: 25 μm (applies to all images).

To assess whether these changes in focal adhesion phenotypes also correlated with changes in cytoskeletal organization, the actin cytoskeleton was visualized with phalloidin (Fig 7A). We observed RPTECs plated on 4 Pa and 0.1 kPa gels predominantly displayed peripherally distributed actin. As substrate stiffness increased, a larger fraction of cells displayed central stress fibers spanning the cell cytoplasm. We again observed significant heterogeneity in the RPTEC population even on glass substrates, compared to NHLFs that uniformly display central stress fibers. Semiquantitatively classifying the actin cytoskeletal phenotype as predominantly peripheral (0–10 central stress fibers), indeterminate (11–50), or central (51+) again showed a shifting of RPTEC phenotype with increasing substrate stiffness (Fig 7B). We noted the fraction of cells showing central stress fibers was generally greater than the fraction of cells showing robust vinculin containing focal adhesions. After 4 d, we found similar distributions of both vinculin-containing focal adhesions and actin stress fibers (S4 Fig).

### Stiffness promotes FAK and ERK activation

**Fig 8:**
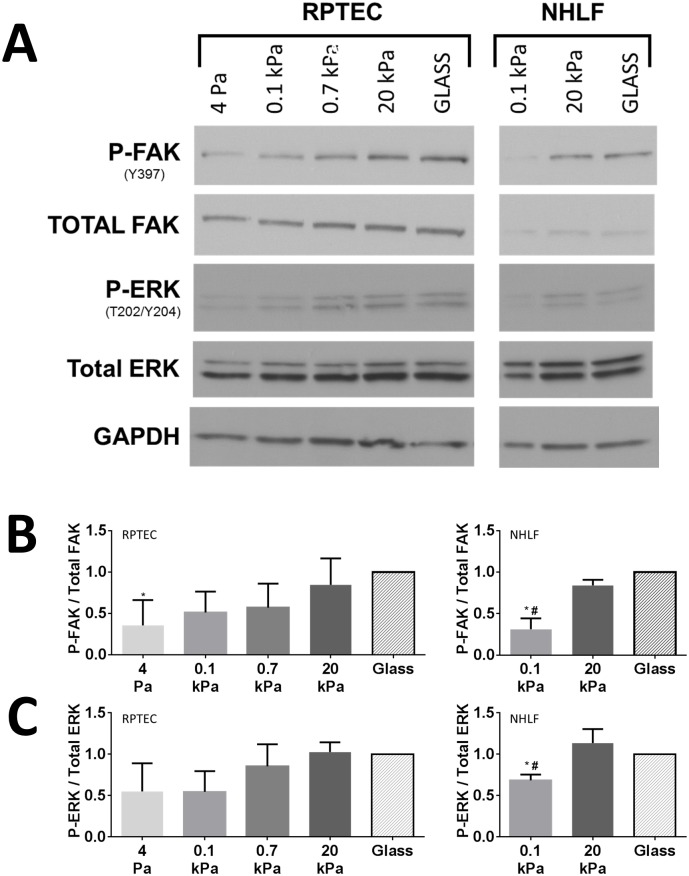
Increasing substrate stiffness promotes early FAK autophosphorylation and ERK 1/2 activation. (A) Representative western blots for phospho-FAK (Y397, P-FAK), total FAK, phospho-ERK 1/2 (T202/Y204, P-ERK), total ERK 1/2, and GAPDH for RPTECS (left) and NHLFs (right) 24 h after plating. (B) Densitometry for relative P-FAK per total FAK for RPTEC (left, N = 5) and NHLF experiments (right, N = 3). (C) Densitometry for relative P-ERK/Total ERK for RPTEC (left, N = 3) and NHLF experiments (right, N = 3). *: *p* < 0.05 relative to glass and #: *p* < 0.05 relative to 20 kPa and glass for multiple comparisons by ANOVA with Tukey’s *post-hoc* test. ANOVA with *post-hoc* trend analyses for P-FAK/Total FAK and P-ERK/Total ERK were highly significant (*p* = 0.0003 and *p* = 0.002, respectively, for trend from 4 Pa to Glass).

Given that FAK activation has been linked to substrate stiffness and cytoskeletal organization in other cell types [32], we assessed the stiffness dependence of autophosphorylation of FAK at Y397 in RPTECs as an alternative way to validate our semiquantitative immunofluorescence findings. The relative ratio of Y397-phosphorylated FAK increased with substrate stiffness (Fig 8A and 8B). ANOVA with *post-hoc* trend analysis for increasing FAK phosphorylation with increasing stiffness was highly significant (*p* < 0.001). After 4 d of culture, differences in FAK phosphorylation due to substrate stiffness were less prominent (S5 Fig). We noted that the relative differences in FAK phosphorylation due to substrate stiffness were similar for RPTECs and NHLF controls (Fig 8B).

**Fig 9:**
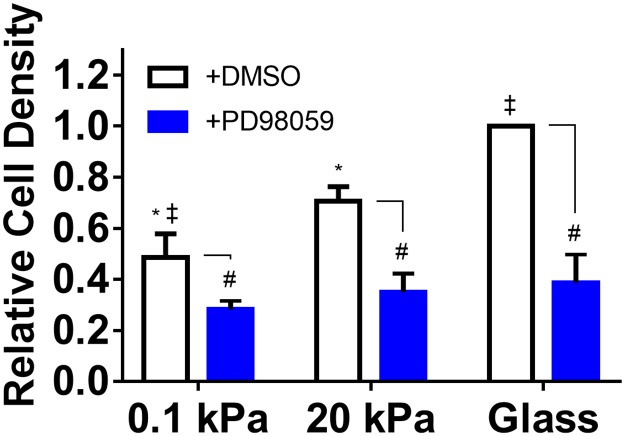
ERK activation is required to mediate the effects of stiffness on RPTEC proliferation. RPTECs were cultured on hydrogel substrates in the presence of 50 μM PD98059 or DMSO vehicle (exchanged daily) for 4 d. DAPI stained nuclei were quantified and normalized to RPTEC density on glass with vehicle. *: *p* < 0.05 relative to glass; ‡: *p* < 0.05 relative to 20 kPa; #: *p* < 0.05 relative to vehicle control. There were no statistical differences in cell density between conditions treated with PD98059.

As we and others have previously reported correlations between FAK and ERK activation in response to ECM stiffness cues [11,33], and the role of ERK 1/2 in mediating cell proliferation has been clearly established (reviewed [34]), we assessed if ERK 1/2 activation was similarly modulated by ECM stiffness in our model. We found a pattern of ERK phosphorylation at T202/Y204 after 1 d of culture that very closely mirrored the pattern of activation observed for FAK (Fig 8A and 8C). The relative differences in ERK 1/2 phosphorylation due to substrate stiffness were similar between RPTECs and NHLFs (Fig 8C). We further established that PD98059, an inhibitor of ERK activation, attenuates RPTEC proliferation at a minimum concentration of 50 μM and that this results in decreased ERK phosphorylation over a 4 d culture period (S6 Fig). Using this concentration to inhibit ERK activation in RPTECs on hydrogel substrates, we found PD98059 significantly decreased cell density after 4 d of culture and also attenuated the effects of stiffness (Fig 9), indicating that ERK activation is required to mediate the effect of stiffness on RPTEC proliferation.

### Optimal substrate stiffness enhances epithelialization

**Fig 10:**
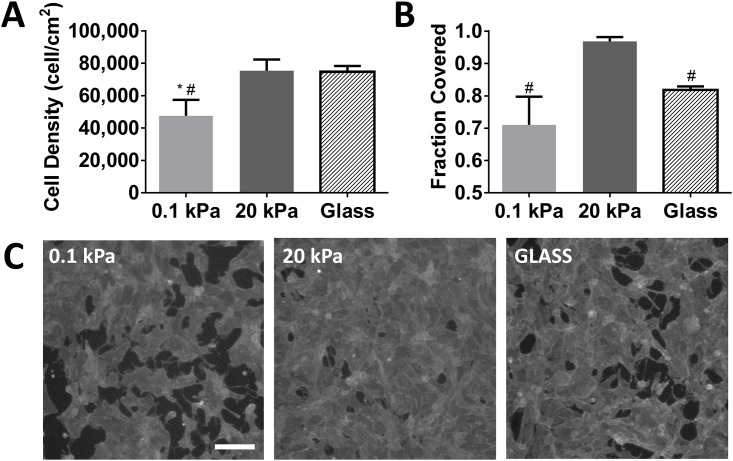
Stiff hydrogels promote enhanced epithelialization independent of proliferation. (A) Cell density determined by automated counting of DAPI stained nuclei after 14 d of culture. (B) epithelialization measured by phalloidin positive area fraction. (C) representative contrast enhanced fluorescent images of phalloidin stained surfaces. Images shown had area fraction covered closest to the group mean. *: *p* < 0.05 relative to glass; #: *p* < 0.05 relative to 20 kPa. Scale bar: 200 μm (applies to all images).

In addition to evaluation of short-term cell behavior, we also assessed the effects of substrate stiffness on long-term differences in cell proliferation and epithelial coverage. We observed that cell proliferation on softer substrates continued beyond 4 d but never reached the levels observed on the stiffer gels or glass (Fig 10A). Not surprisingly, this lower cell density correlated with innumerable large defects in the epithelial layer (Fig 10B and 10C). The stiffer hydrogel substrates demonstrated the development of a near perfect confluent layer of cells that covered greater than 96% of the culture area. Surprisingly, though cell density on glass was similar to the stiff gels, RPTECs tended to aggregate into dense clusters, leaving significant defects in the epithelial layer, suggesting that optimal stiffness may be needed to balance cell proliferation with maturation of a confluent epithelium.

**Fig 11:**
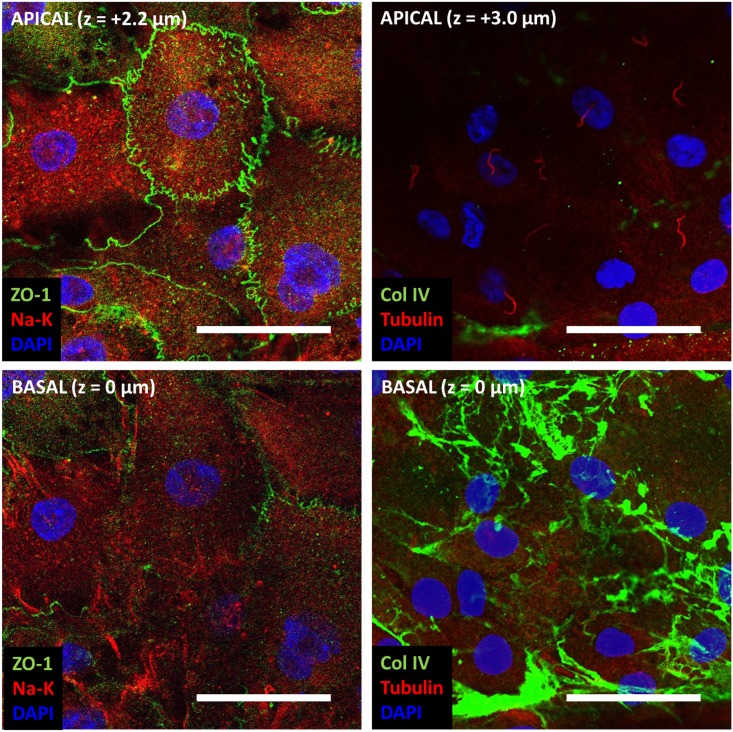
RPTECs on stiff hydrogels develop tight junctions and show polarity. RPTECs cultured for 14 d on 20 kPa gels were fixed and stained for the tight junction protein zonula occludens-1 (ZO-1, green, left column), sodium potassium ATPase (Na-K, red, left column), collagen IV (Col IV, green, right column), and acetylated tubulin (Tubulin, red, right column, highlights the central cilium). Nuclei were counterstained with DAPI (blue). Each column shows confocal microscopy images from the same x-y position. The bottom row shows staining at the basal aspect of the cells and the top row shows staining at the apical aspect of the cells (z-position as indicated). Scale bar: 50 μm.

To characterize these RPTEC monolayers, we examined the expression and localization of several markers of epithelial phenotype after 14 d of culture. We found localization of the tight junction protein zonula occludens-1 (ZO-1) at the apical aspect of cell-cell junctions and more robust staining of sodium potassium (Na-K) ATPase at the basal aspect of the cells (Fig 11, S7 Fig, S1 Movie) suggesting polarization. Though ZO-1 expression was not observed at the edges of defects (i.e. areas with no cell-cell interactions that were more common on the 0.1 kPa and glass substrates), we did appreciate these characteristics in areas of confluence regardless of substrate stiffness (Fig 11, S7 Fig). Confocal z-stacks demonstrated that RPTECs on these substrates tended to be relatively flat (S1 Movie). We rarely observed lateral Na-K ATPase localization. We also noted that all cells had a single acetylated tubulin containing central cilium uniformly oriented apically (Fig 11, S7 Fig). RPTECs organized a fibrillar collagen IV basement membrane on their basal aspect with far more complexity than the collagen IV deposited on the substrates prior to cell seeding (Fig 11, S7 Fig compared with S1 Fig). These findings strongly support an epithelial phenotype of the RPTEC monolayers with tight junction formation, polarization, and organized basement membrane formation.

Given that epithelial-to-mesenchymal transition has been associated with substrate stiffness [29], we also examined the expression of the mesenchymal marker alpha smooth muscle actin. After 14 d, we found expression to be similar regardless of substrate and substantially less abundant than in fibroblast controls (S8A and S8B Fig). After 14 d, RPTECs also expressed gamma-glutamyltransferase, which is widely expressed but undergoes tissue-specific post-translational modification [35] (manifested as different banding patterns on electrophoresis), with an electrophoresis banding pattern that was similar to human kidney lysate, did not vary with substrate stiffness, and was distinct from fibroblast controls (S8C and S8D Fig).

## Discussion

In this study, we report an *in vitro* model using a biocompatible scaffold system with human RPTECs to explore the role of ECM stiffness in recovery from acute kidney injury. We developed and characterized a versatile PEG-based scaffold platform with well-controlled substrate mechanics, which were independent of ECM conjugation. Using this well-characterized platform, we observed that increasing substrate stiffness promotes spreading and proliferation of RPTECs. We then showed cytoskeletal organization and phosphorylation of FAK, but not YAP shuttling or apoptosis, was correlated with proliferation and that ERK 1/2 activation was required to mediate this effect. We also showed that substrate stiffness contributed to optimal RPTEC epithelialization.

The platform developed here has several advantages. Many studies of substrate mechanics, including those to date with renal tubular epithelium [17,20], utilize natural ECMs that are modified by varying composition or crosslinking. While this approach has the potential to recapitulate a more natural ECM environment, it is difficult to disentangle the effects of mechanics from the biochemical changes used to induce differences in mechanical properties (e.g. chemical crosslinking, increased concentration, changes in constituents). The range of achievable mechanical properties also is limited. While polyacrylamide hydrogels have been used widely as a synthetic substitute to overcome these limitations [17,36], the inherent toxicity of the precursors limits their use for regenerative medicine. PEG-based hydrogel systems, as presented here, also have well-defined properties and permit orthogonal control of cell-matrix interactions and mechanics, but are non-toxic and have the potential to be formed in the presence of living cells [37]. In addition to these features, PEGDA-based hydrogels have been formed into a variety of geometric configurations including microfluidic devices [38] and micro-[39] or nano-[40] patterned substrates. PEG systems also have been rendered proteolytically degradable using several approaches [41–43]. This versatility makes PEG scaffolds useful for renal regenerative strategies [21] and allows our findings to be readily incorporated into the design of such systems.

Using this platform, we generated synthetic ECMs with moduli spanning 4 orders of magnitude. Though we observed the most extreme effects on 3.50% PEGDA, we also observed substantial variability in the properties of these hydrogels, likely because their composition was near the gelation threshold for the system. Also, given that the moduli of these gels (4 ± 2 Pa) is similar to mucus and unlikely to be of relevance in the kidney, we focused on comparing 4.25% PEGDA (0.13 ± 0.03 kPa) and 10.0% PEGDA (19 ± 0.6 kPa). These substrates span 2 orders of magnitude and are in a range relevant to kidney tissues [9,44].

While bulk measurements of the renal parenchyma clearly demonstrate stiffening in CKD [9], whether these changes can be sensed by renal tubular epithelial cells remains an unanswered question. Differences in stiffness in a mouse model of CKD, determined by direct AFM measurement of mouse kidney tissues before or after ureteral outlet obstruction, have been reported that are similar to the moduli reported here [17]. Furthermore, the thickness of the tubular basement membrane, even in diabetic CKD where thickening is a hallmark of disease, is generally less than 1 μm [45]. Traction force microscopy in 3 dimensions indicates that cell traction forces penetrate at least 10 times deeper [46], suggesting that changes in the bulk stiffness of the parenchyma likely can be sensed by the tubular epithelium. Also, in other tissues, nanoscale stiffness measurements of the basement membrane using atomic force microscopy have been correlated with macroscale mechanical parameters [47]. These reports suggest that our model likely is relevant to the microenvironment of the renal tubule.

In the last decade, several major paradigms for how cells use mechanics to decide between death, differentiation, and growth have emerged in which extracellular stiffness, intracellular tension, and cell size are central determinants of these effects. Early after plating, we observed differences in stiffness dependent cell size. Cell size, which is related to the balance of intracellular tension and extracellular stiffness, has been correlated with cell proliferation [48]. However, matrix stiffness [29,49] and cell size specifically [50] also have been correlated with apoptosis, which could have explained the observed differences in cell density. However, we did not observe evidence of stiffness-dependent differences in apoptosis in RPTECs. More recently, YAP nuclear localization also has been correlated with cell size and substrate stiffness-dependent regulation of cell proliferation [51]. We found RPTECs largely displayed nuclear YAP and that this was relatively insensitive to substrate stiffness after 1 d, compared with MSCs, which showed a significant stiffness dependence (though attenuated compared with other reports [18]). YAP localization was completely insensitive to substrate stiffness after 2 d. These data suggest YAP nuclear localization was not the driving mediator of differences in RPTEC proliferation.

In contrast, we observed a clear correlation between substrate stiffness and focal adhesion development, cytoskeletal organization, and FAK activation. Our results suggest that these interdependent pathways are critical for substrate stiffness permissive proliferation in RPTECs, as has been described in many other cell types (reviewed [32]). We also showed that stiffness mediated proliferation is correlated with ERK activation and that pharmacologic inhibition of ERK activation significantly attenuates the effects of stiffness on proliferation. Cytoskeletal organization and, in particular, FAK activation have been recognized as important upstream mediators of ERK activation. (reviewed [52]). How these pathways interact with other drivers of cell proliferation is becoming increasingly better understood. Of particular interest, substrate stiffness sensitizes epithelial cell lines to epidermal growth factor [53], which was a key driver of RPTEC proliferation in our model, and is important during recovery from AKI [54]. While the mechanisms we report are not new, our demonstration that they are preserved in primary human RPTECs is an important contribution that strengthens the link between mechanistic studies conducted with epithelial cell lines and human pathophysiology.

We further observed that these signaling changes, which were most prominent very early in culture, were correlated with epithelialization 2 weeks later. We observed that RPTECs on stiff gel substrates achieved a near perfect monolayer, an essential requirement for epithelial function. While it is interesting that RPTECs did not achieve confluent monolayers on very stiff glass controls, the fundamental dissimilarities in surface chemistry make it difficult to attribute this difference to mechanics definitively. However, the results do suggest that RPTEC proliferation alone is not sufficient to achieve confluence. Aside from surface coverage, we did not identify any other substantial difference in RPTEC phenotype at this stage. RPTECs on all substrates in areas of confluence showed evidence of tight junction formation and rudimentary polarity but remained flat, unlike mature cuboidal renal epithelium *in vivo*. There also was no evidence of stiffness mediated differences in epithelial-to-mesenchymal transition at this stage, which has been implicated in stiffness dependent effects in other studies [17,29]. Expression of GGT protein, a marker of RPTEC phenotype, was similar to that of human kidney extract and did not vary with substrate stiffness. Together these findings suggest that stiffness may modulate the initial stages of recovery from AKI characterized by restoration of epithelial coverage of the denuded basement membrane but is not sufficient to promote re-differentiation of RPTECs toward a mature functional renal tubular epithelium.

Our work complements that of the Tang group [17,20,49], who have investigated the effects of matrix mechanics in renal cell lines and primary mouse RPTECs. Using a distinctly different, well-defined substrate system based on PEGDA hydrogels, we have shown that, like mouse RPTECs on polyacrylamide or natural ECM materials, RPTECs of human origin have enhanced proliferation as substrate stiffness increases and extended these findings to show that these early changes in proliferation are linked to longer-term differences in epithelial coverage. In addition, our hydrogel platform was coated with collagen IV, rather than collagen I, suggesting an important role of substrate stiffness irrespective of ECM ligand. We also have shown that the effects of stiffness on proliferation are associated with activation of ERK 1/2 and have further correlated changes in cytoskeletal organization and FAK activation with this finding. Overall, these findings suggest a similarity between rodent and human RPTEC behavior and strengthen the hypothesis that ECM mechanics are important modulators of RPTEC behavior.

Considering the poor outcomes in CKD patients after AKI and increased stiffness in CKD kidneys, we initially hypothesized that stiffness may, in fact, retard RPTEC epithelialization. Taken together, our *in vitro* model findings do not support this hypothesis and suggest instead that increased ECM stiffness may serve as a mitigating factor that facilitates recovery of the limited number of surviving nephrons after AKI by providing a permissive environment for cell proliferation. There also is *in vivo* evidence the mechanotransduction pathways implicated in our model play an important role in recovery from AKI. After ischemia-reperfusion injury, focal adhesion and cytoskeletal reorganization have been observed, and after a brief early rise and return to baseline, phosphorylated FAK and ERK 1/2 begin to rise 24 h later [19]. Mice in which FAK has been conditionally deleted from proximal tubular epithelial cells just prior to ischemia-reperfusion injury have decreased cell proliferation 24 h after injury [55]. It has also been noted that epidermal growth factor, which signals in part through ERK, is critically important for recovery from ischemia-reperfusion AKI in mice [54]. Together these studies support the hypothesis that factors that modulate the FAK-ERK signaling pathway, such as substrate mechanics as demonstrated here, may modulate the recovery process after AKI.

Though much attention has been given to the role of stiffness in promoting epithelial-to-mesenchymal transition along with its potential deleterious consequences [20,29], our work highlights the potential positive effects of stiffness mediated changes in RPTEC phenotype after AKI. While stiffness may negatively affect the response of cells in the interstitium, fate mapping studies suggest that renal epithelial cells do not migrate into this environment [56]. In fact, epithelial-mesenchymal-epithelial cycling actually may be central to the regenerative process [57]. For the renal epithelium, our results suggest that stiffness may have an important role in the first phase of this cycle, but what role ECM stiffness plays on mesenchymal-to-epithelial redifferentiation, and, more broadly, if these effects have physiologic relevance in the complex milieu present after AKI, remain questions for future investigation.

## Supporting information

S1 Table Antibodies used in immunofluorescence and western blot experiments. (PDF)

S1 Fig Detailed analysis of ECM protein conjugation. (A) Representative images chosen with similar relative intensities for various substrates as acquired and quantified (top row) and with contrast enhancement (using the autocontrast feature of ImageJ) to highlight surface modification (bottom row). (B) Log transformed histogram of pixel intensities of original images showing similar change in distribution (relative to controls, dashed lines) similar to that presented by Wen *et al*. for polyacrylamide gels [28]. Percentages indicate composition of PEGDA at polymerization (% w/w) for hydrogel substrates. Scale bar: 100 μm (applies to all images).
(TIF)

S2 Fig DAPI stained nuclei do not show evidence of nuclear fragmentation 24h after plating. Representative images are shown. Bottom right image shows cells treated with 2 μM staurosporine for 24 h as a reference. Yellow arrows indicate cells with evidence of nuclear fragmentation. Scale bar: 100 μm.
(TIF)

S3 Fig YAP is uniformly localized to the nucleus after 2 d regardless of substrate. Representative images of immunofluorescence staining for YAP in RPTECs after 2 d of culture. Review of all images did not review any cells with YAP not predominantly localized to the nucleus, observed in 2 independent experiments. Scale bar: 200 μm (applies to all images).
(TIF)

S4 Fig Distribution of vinculin-containing focal adhesions and stress fibers in RPTECs after 4 d. (A) Focal adhesions (FA) were identified using immunofluorescent staining for vinculin (grayscale). (B) Filamentous actin central stress fibers (SF) were identified with fluorescently tagged phalloidin (grayscale). Randomly selected cells were categorized as described for Figs 6 and 7. χ^2^ contingency table analysis was highly significant for substrate effect on distribution, *p* < 0.0001 for both vinculin and stress fibers.
(TIF)

S5 Fig FAK phosphorylation is less dependent on substrate stiffness at 4 d. (A) Representative western blots for phospho-FAK (Y397, P-FAK), total FAK, and GAPDH for RPTECS after 4 d of culture. (B) Densitometry for relative P-FAK per total FAK for RPTECs.
(TIF)

S6 Fig PD98059 inhibits RPTEC proliferation and ERK activation. (A) RPTECs were seeded on tissue culture plastic and cultured in REGM in the presence of various concentrations of PD98059 or DMSO vehicle (made fresh and changed daily). After 4 d, cell density was determined by counting DAPI stained nuclei and normalized to RPTECs cultured in unmodified REGM. (B) Representative western blots of phospho-ERK 1/2 (T202/Y204, P-ERK), total ERK 1/2, and GAPDH at 4 d for RPTECS cultured in REGM or REGM supplemented with 50 μM PD98059 or DMSO as indicated. (C) Densitometry for relative P-ERK / total ERK (N = 3). *: *p* < 0.05 compared with REGM or +DMSO (and in subpanel A, 1 μM or 10 μM PD98059).
(TIF)

S7 Fig RPTECs on soft hydrogels and glass also develop tight junctions and show polarity. RPTECs cultured for 14 d on 0.1 kPa gels and glass were fixed and stained for the tight junction protein zonula occludens-1 (ZO-1, green, left column), sodium potassium ATPase (Na-K, red, left column), collagen IV (Col IV, green, right column), and acetylated tubulin (Tubulin, red, right column, highlights the central cilium). Nuclei were counterstained with DAPI (blue). For each substrate, a pair of images are shown from the same x-y position. Rows 1 and 3 show staining at the apical aspect of the cells and rows 2 and 4 show staining at the basal aspect of the cells (z-position as indicated). Scale bar: 50 μm (applies to all images).
(TIF)

S8 Fig RPTEC expression of mesenchymal and epithelial markers is distinct from NHLFs. Expression patterns in RPTECs did not vary with substrate stiffness and were distinct from fibroblasts (used as a prototypical mesenchymal cell). (A) Representative western blots and (B) associated densitometry (normalized to GAPDH then to fibroblast controls, N = 3) of expression of alpha smooth muscle actin (SMA, used as a marker of mesenchymal phenotype) in RPTECs after 14 d of culture on various substrates compared with NHLFs (cultured routinely on tissue culture plastic). (C) Representative western blots showing immunoreactive banding patterns for gamma-glutamyltransferase (GGT, see S1 Table for antibody information) in RPTECs after 14 d of culture compared with NHLFs or human whole kidney lysate and (D) associated densitometry (normalized to GAPDH then to fibroblast controls, N = 3) for selected bands.
(TIF)

S1 Movie RPTECs on stiff hydrogels develop tight junctions and show polarity. 3D rendering of a Z-stack of ZO-1 (green) and Na-K ATPase (red) staining of RPTECs cultured on 20 kPa hydrogels for 14 d.
(MPG)
