# Supplementary material for: Engineered extracellular matrices with controlled mechanics modulate renal proximal tubular cell epithelialization
Source: PLoS One. 2017 Jul 17;12(7):e0181085. doi: 10.1371/journal.pone.0181085 (PMC5513452; doi:10.1371/journal.pone.0181085)
Supplement: S1 Table — (PDF) [file pone.0181085.s001.pdf]

Antibody Target	MW[#] (kD)	Clone	Species	Dilution	Application	Manufacturer	Catalog Number
Acetyl-alpha-Tubulin	N/A	6-11B-1	Mouse IgG2b	1:400	IF	TFS[*]	32-2700
Collagen IV	N/A	COL-94	Mouse IgG1	1:400	IF	TFS	MA1-22148
Vinculin	N/A	hVIN-1	Mouse IgG1	1:400	IF	Sigma	V9131
YAP	N/A	63.7	Mouse IgG2a	1:200	IF	SCBT	101199
ZO-1	N/A	ZO1-1A12	Mouse IgG1	1:400	IF	TFS	33-9100
Smooth Muscle Actin	42	1A4	Mouse IgG 2a	1:1000	WB	TFS	MA5-11547
Cleaved Caspase 3	17, 19	5A1E	Rabbit IgG	1:1000	WB	CST	9664
ERK1/2	44, 42	137F5	Rabbit IgG	1:1000	WB	CST	4695
FAK	125	D2R2E	Rabbit IgG	1:1000	WB	CST	13009
GGT	Multiple[&]	3E6	Mouse IgG 2a	1:1000	WB	SCBT	100746
GAPDH	37	Polyclonal	Goat	1:2000	WB	SCBT	20357
Phospho ERK1/2 (T202/Y204)	44, 42	D13.14.4E	Rabbit IgG	1:2000	WB	CST	4370
Phospho FAK (Y397)	125	D20B1	Rabbit IgG	1:1000	WB	CST	8556

Abbreviations:
CST-Cell Signaling Technology, Danvers, MA
ERK1/2-Extracellular signal regulated kinase (p44/42 mitogen-activated protein kinase)
FAK-Focal adhesion kinase
GGT-gamma-glutamyltransferase
GAPDH-glyceraldehyde 3-phosphate dehydrogenase
IF-Immunofluorescence
SCBT-Santa Cruz Biotechnology, Dallas, TX
TFS-Thermo Fisher Scientific, Waltham,MA
WB-Western blot
YAP-Yes-associated protein
ZO-1-Zonula occludens-1

[#]molecular weight used to identify relevant band(s) in all reported western blot images.
[*]was received as a kind gift from Innovative Biotherapies, Ann Arbor, MI.
[&]see text for details
