# Supplementary figures and images for: Engineered extracellular matrices with controlled mechanics modulate renal proximal tubular cell epithelialization
Source: PLoS One. 2017 Jul 17;12(7):e0181085. doi: 10.1371/journal.pone.0181085 (PMC5513452; doi:10.1371/journal.pone.0181085)

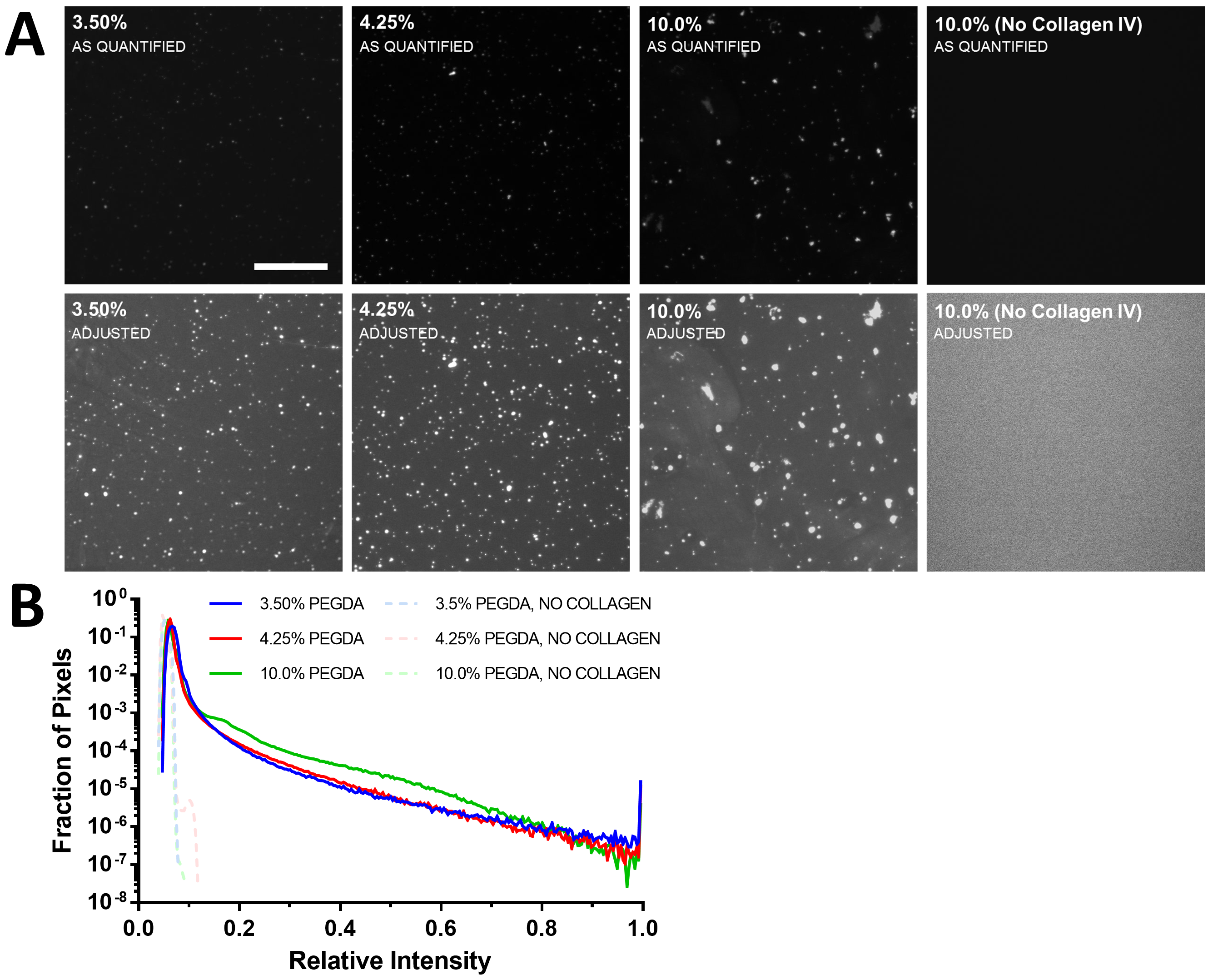

Supplement: S1 Fig — (A) Representative images chosen with similar relative intensities for various substrates as acquired and quantified (top row) and with contrast enhancement (using the autocontrast feature of ImageJ) to highlight surface modification (bottom row). (B) Log transformed histogram of pixel intensities of original images showing similar change in distribution (relative to controls, dashed lines) similar to that presented by Wen et al. for polyacrylamide gels [28]. Percentages indicate composition of PEGDA at polymerization (% w/w) for hydrogel substrates. Scale bar: 100 μm (applies to all images). (TIF) [file pone.0181085.s002.tif]

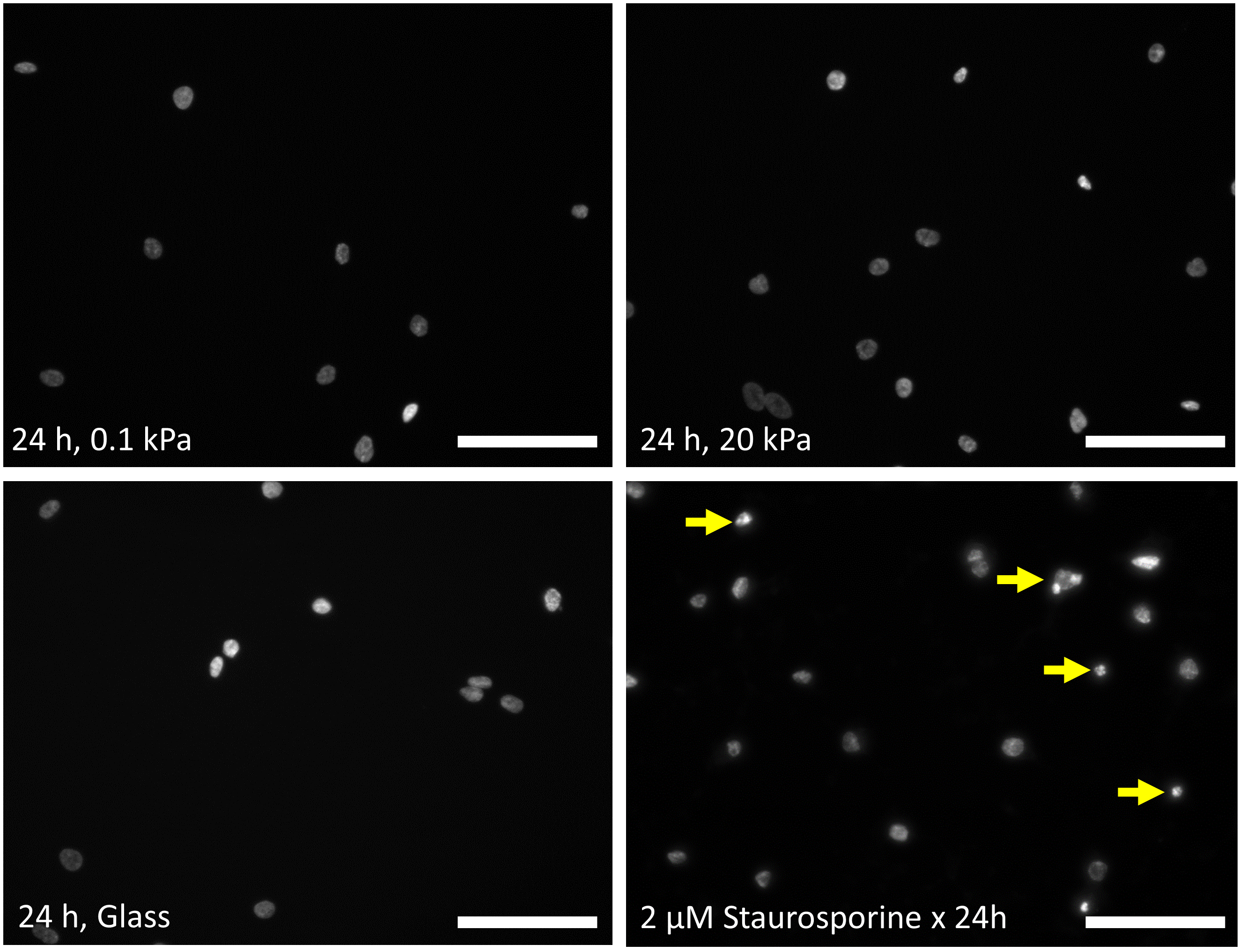

Supplement: S2 Fig — Representative images are shown. Bottom right image shows cells treated with 2 μM staurosporine for 24 h as a reference. Yellow arrows indicate cells with evidence of nuclear fragmentation. Scale bar: 100 μm. (TIF) [file pone.0181085.s003.tif]

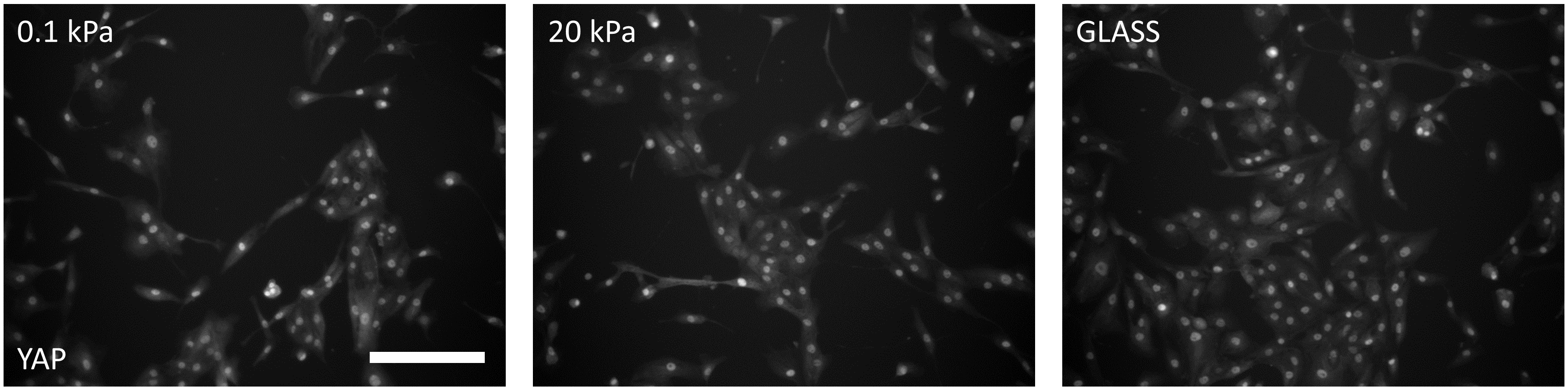

Supplement: S3 Fig — Representative images of immunofluorescence staining for YAP in RPTECs after 2 d of culture. Review of all images did not review any cells with YAP not predominantly localized to the nucleus, observed in 2 independent experiments. Scale bar: 200 μm (applies to all images). (TIF) [file pone.0181085.s004.tif]

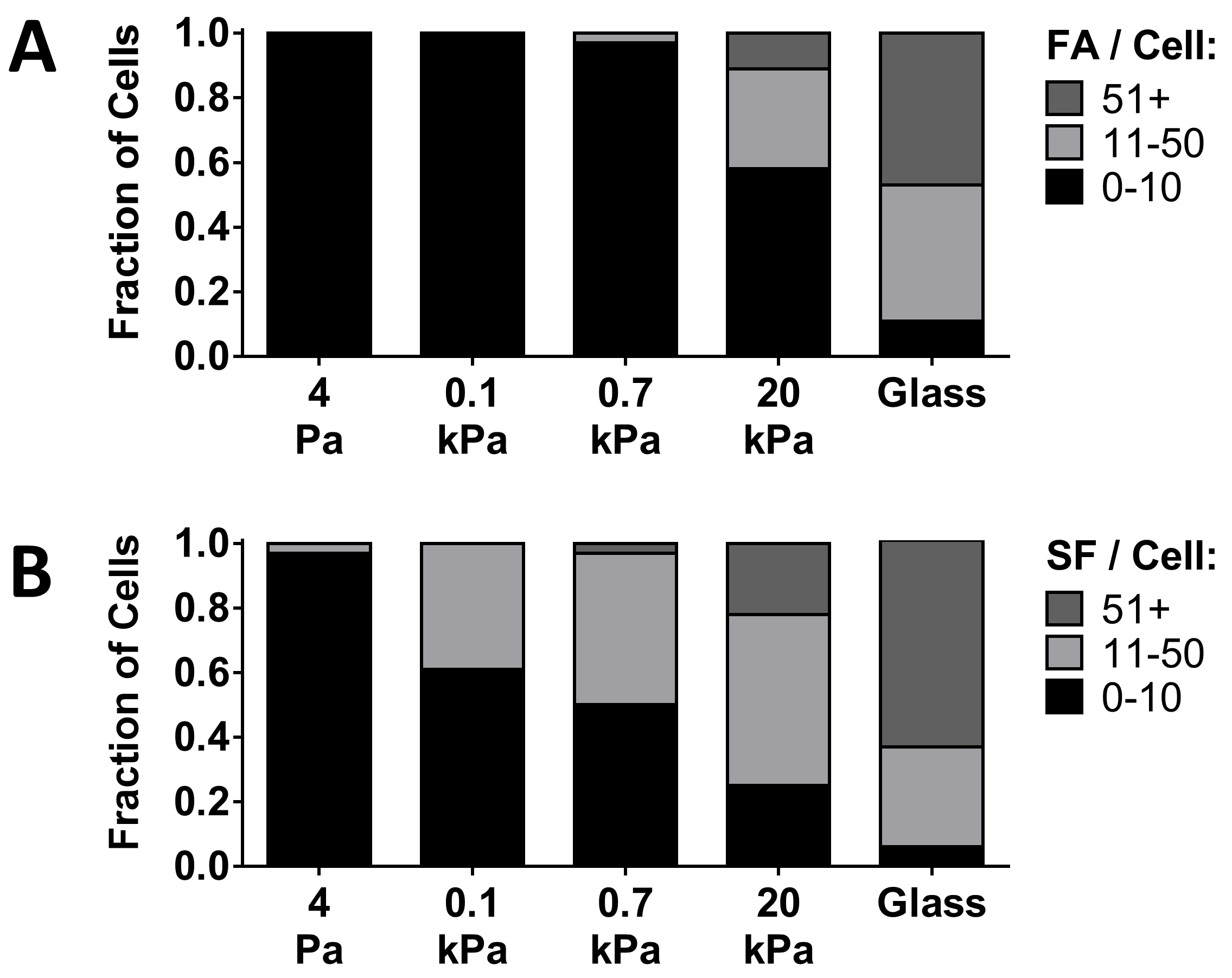

Supplement: S4 Fig — (A) Focal adhesions (FA) were identified using immunofluorescent staining for vinculin (grayscale). (B) Filamentous actin central stress fibers (SF) were identified with fluorescently tagged phalloidin (grayscale). Randomly selected cells were categorized as described for Figs 6 and 7. χ2 contingency table analysis was highly significant for substrate effect on distribution, p < 0.0001 for both vinculin and stress fibers. (TIF) [file pone.0181085.s005.tif]

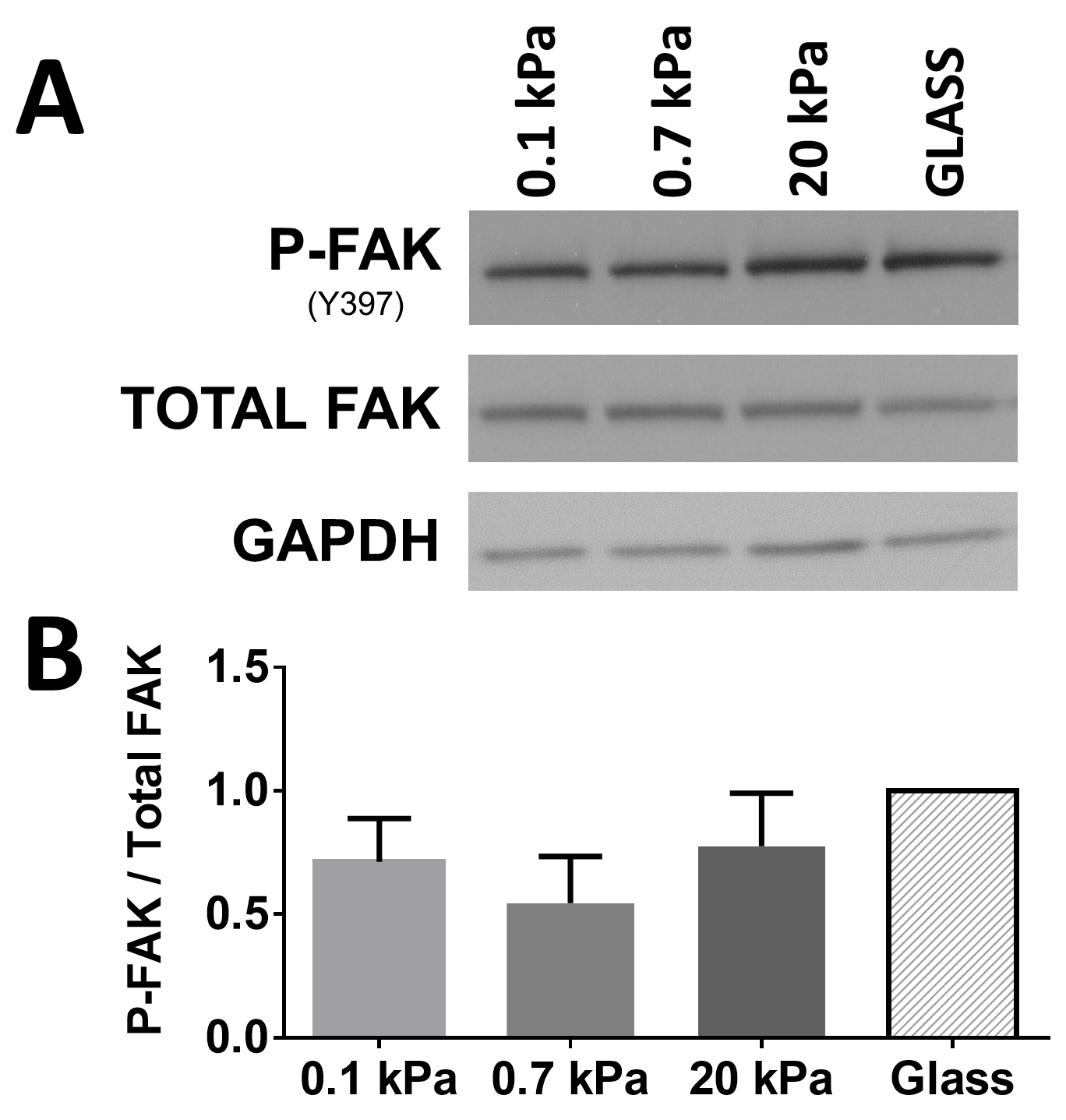

Supplement: S5 Fig — (A) Representative western blots for phospho-FAK (Y397, P-FAK), total FAK, and GAPDH for RPTECS after 4 d of culture. (B) Densitometry for relative P-FAK per total FAK for RPTECs. (TIF) [file pone.0181085.s006.tif]

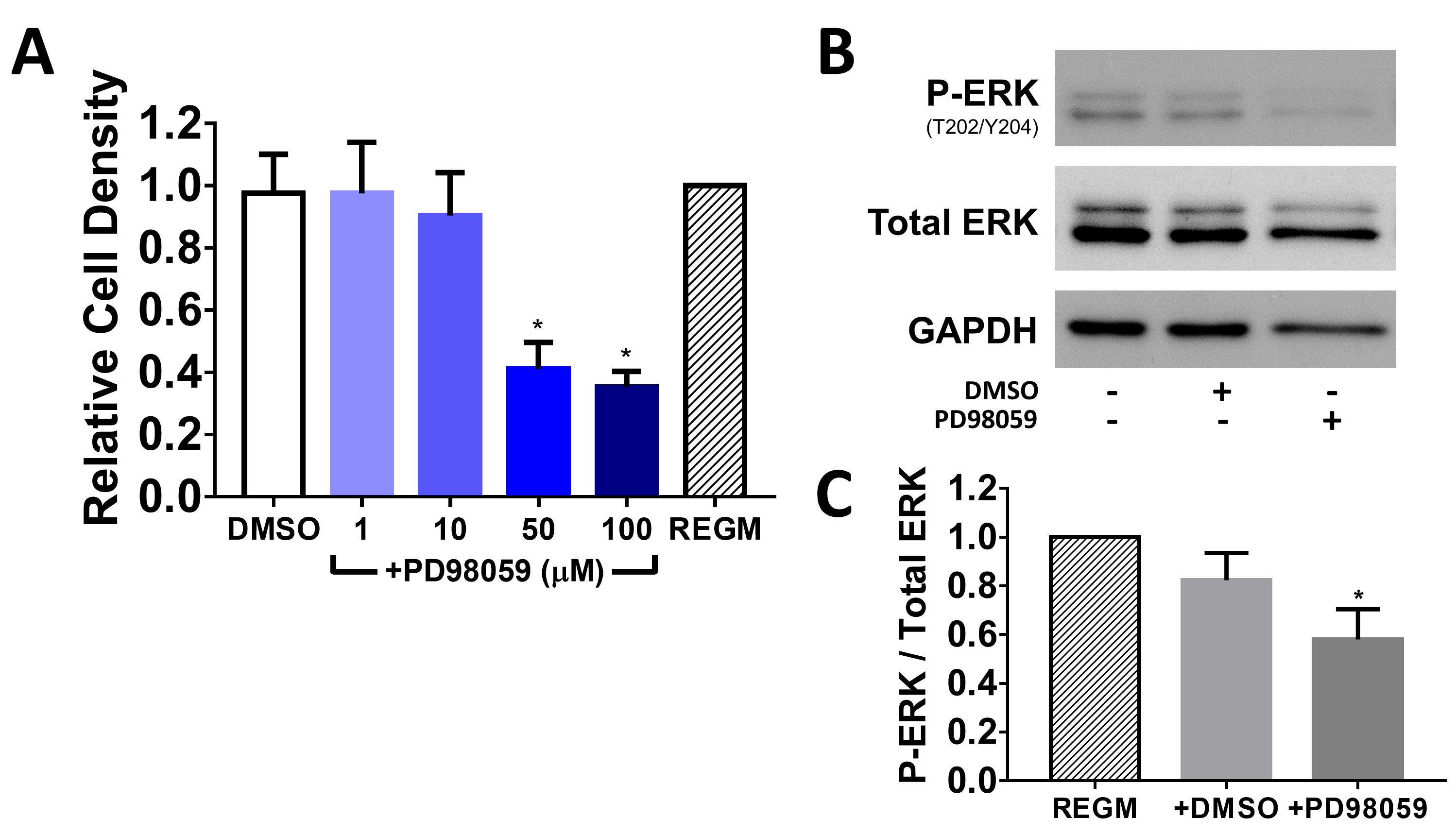

Supplement: S6 Fig — (A) RPTECs were seeded on tissue culture plastic and cultured in REGM in the presence of various concentrations of PD98059 or DMSO vehicle (made fresh and changed daily). After 4 d, cell density was determined by counting DAPI stained nuclei and normalized to RPTECs cultured in unmodified REGM. (B) Representative western blots of phospho-ERK 1/2 (T202/Y204, P-ERK), total ERK 1/2, and GAPDH at 4 d for RPTECS cultured in REGM or REGM supplemented with 50 μM PD98059 or DMSO as indicated. (C) Densitometry for relative P-ERK / total ERK (N = 3). *: p < 0.05 compared with REGM or +DMSO (and in subpanel A, 1 μM or 10 μM PD98059). (TIF) [file pone.0181085.s007.tif]

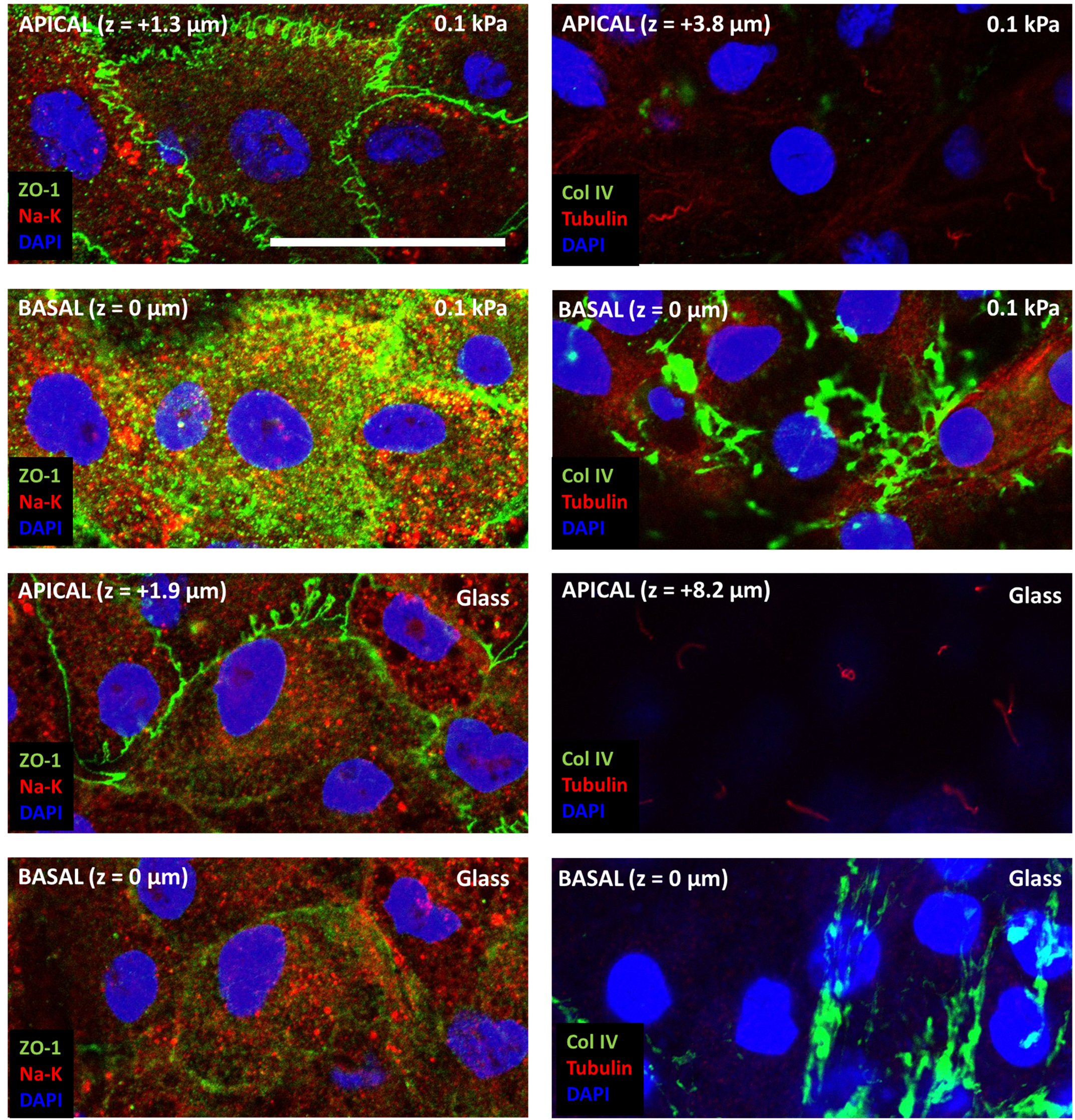

Supplement: S7 Fig — RPTECs cultured for 14 d on 0.1 kPa gels and glass were fixed and stained for the tight junction protein zonula occludens-1 (ZO-1, green, left column), sodium potassium ATPase (Na-K, red, left column), collagen IV (Col IV, green, right column), and acetylated tubulin (Tubulin, red, right column, highlights the central cilium). Nuclei were counterstained with DAPI (blue). For each substrate, a pair of images are shown from the same x-y position. Rows 1 and 3 show staining at the apical aspect of the cells and rows 2 and 4 show staining at the basal aspect of the cells (z-position as indicated). Scale bar: 50 μm (applies to all images). (TIF) [file pone.0181085.s008.tif]

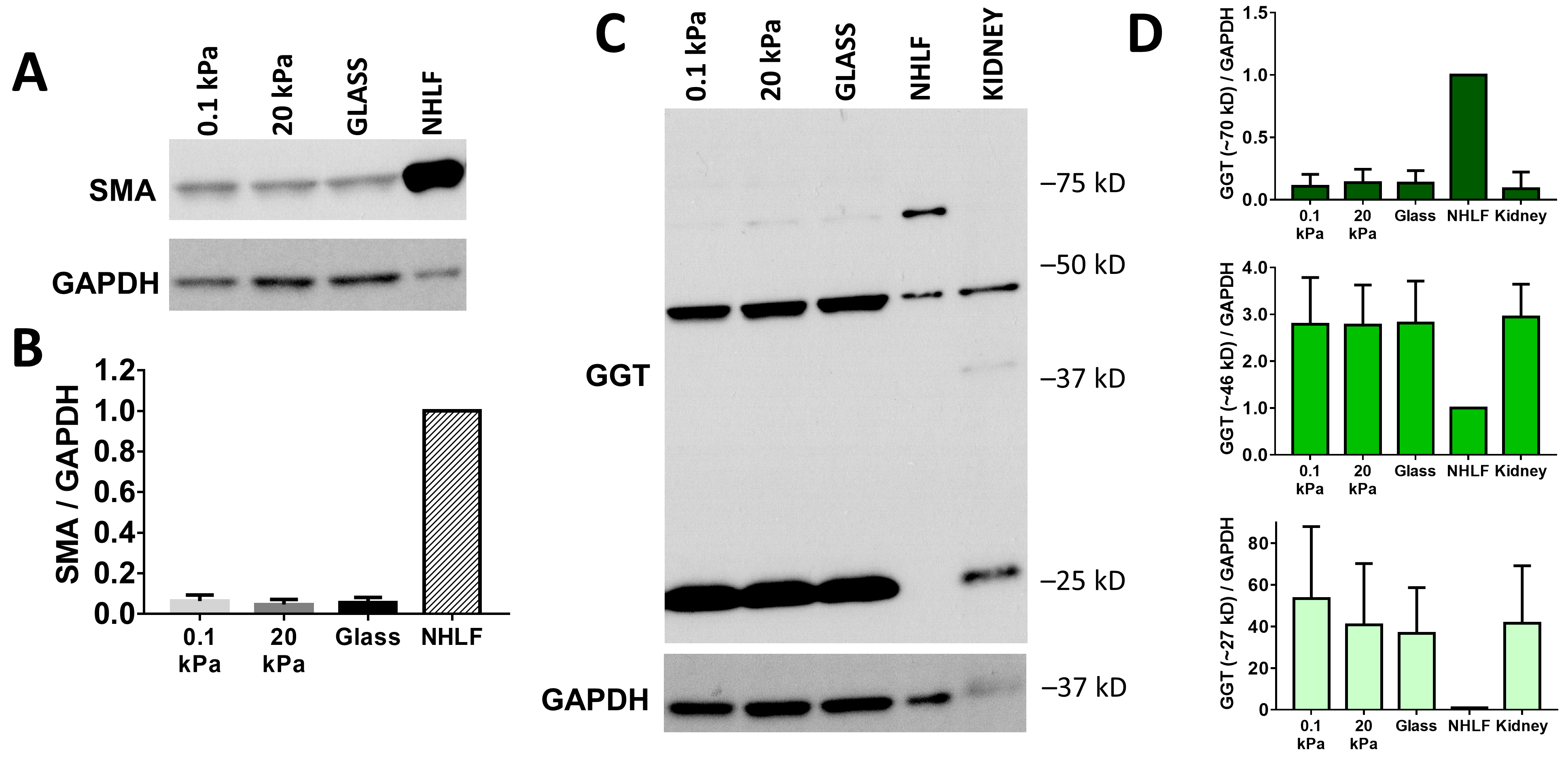

Supplement: S8 Fig — Expression patterns in RPTECs did not vary with substrate stiffness and were distinct from fibroblasts (used as a prototypical mesenchymal cell). (A) Representative western blots and (B) associated densitometry (normalized to GAPDH then to fibroblast controls, N = 3) of expression of alpha smooth muscle actin (SMA, used as a marker of mesenchymal phenotype) in RPTECs after 14 d of culture on various substrates compared with NHLFs (cultured routinely on tissue culture plastic). (C) Representative western blots showing immunoreactive banding patterns for gamma-glutamyltransferase (GGT, see S1 Table for antibody information) in RPTECs after 14 d of culture compared with NHLFs or human whole kidney lysate and (D) associated densitometry (normalized to GAPDH then to fibroblast controls, N = 3) for selected bands. (TIF) [file pone.0181085.s009.tif]
